# Dual Coding Theory Explains Biphasic Collective Computation in Neural Decision-Making

**DOI:** 10.3389/fnins.2017.00313

**Published:** 2017-06-06

**Authors:** Bryan C. Daniels, Jessica C. Flack, David C. Krakauer

**Affiliations:** ^1^ASU–SFI Center for Biosocial Complex Systems, Arizona State UniversityTempe, AZ, United States; ^2^Santa Fe InstituteSanta Fe, NM, United States

**Keywords:** collective computation, decision tasks, critical slowing down

## Abstract

A central question in cognitive neuroscience is how unitary, coherent decisions at the whole organism level can arise from the distributed behavior of a large population of neurons with only partially overlapping information. We address this issue by studying neural spiking behavior recorded from a multielectrode array with 169 channels during a visual motion direction discrimination task. It is well known that in this task there are two distinct phases in neural spiking behavior. Here we show Phase I is a distributed or incompressible phase in which uncertainty about the decision is substantially reduced by pooling information from many cells. Phase II is a redundant or compressible phase in which numerous single cells contain all the information present at the population level in Phase I, such that the firing behavior of a single cell is enough to predict the subject's decision. Using an empirically grounded dynamical modeling framework, we show that in Phase I large cell populations with low redundancy produce a slow timescale of information aggregation through critical slowing down near a symmetry-breaking transition. Our model indicates that increasing collective amplification in Phase II leads naturally to a faster timescale of information pooling and consensus formation. Based on our results and others in the literature, we propose that a general feature of collective computation is a “coding duality” in which there are accumulation and consensus formation processes distinguished by different timescales.

## 1. Introduction

The nervous system is a distributed information processing system. Functional encodings have been identified at the level of single cells (e.g., Shadlen and Newsome, 2001), correlated modules (e.g., Power et al., 2013; Gu et al., 2015), and hemispheres (e.g., Doron et al., 2012). How activity within a scale produces new functional encodings one level up and how the consolidating modules interact to produce coherent, functional behavioral output at the whole brain level are among the primary concerns of cognitive neuroscience (e.g., Gu et al., 2015).

Here we ask how coherent output is produced when neurons in a relevant target population have different “opinions” about an input and are not coordinated by a “Deus Ex Machina” or central controller (e.g., Gazzaniga, 2013). Two competing explanations are supported by the data. One is a “distributed perspective”—coherent output requires encoding the output over many cells (“population-level coding”). The second favors localization—coherent output can be generated by encoding the output by strong activity in one or a few neurons (“grandmother neurons,” reviewed in Gross, 2002, or “sparse coding” Quian Quiroga and Kreiman, 2010).

We show that these two views and the data supporting them can be reconciled by framing the problem of coherent output as one of *collective computation* and drawing on information theory and theories of collective behavior in statistical physics to ask how information from upstream neurons is accumulated and integrated by downstream neurons (whether one or many) and whether the integrated information is disseminated to a broader ensemble.

## 2. Data set and previous work

**Figure 1:**
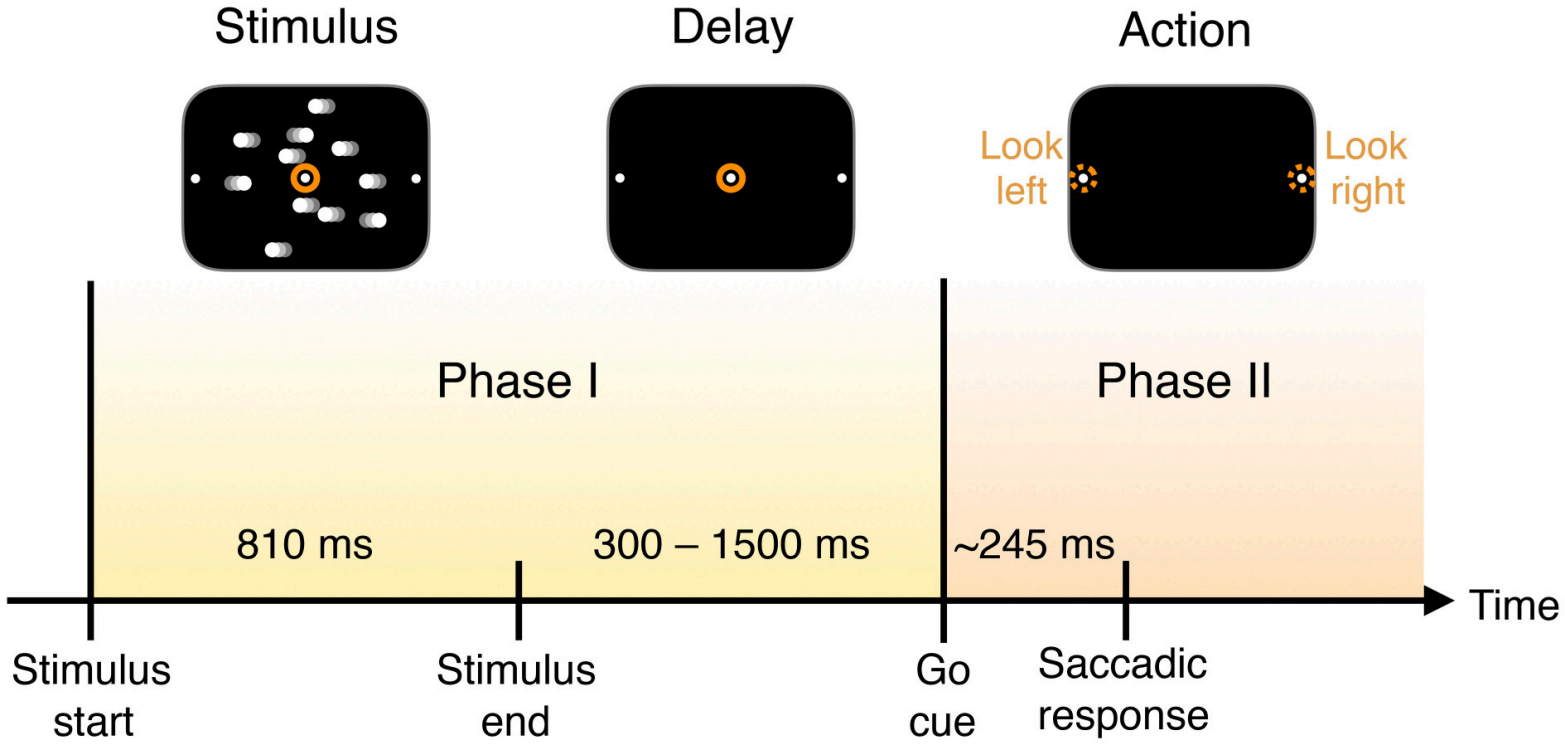
Timing of trial events. A monkey is trained to discriminate opposed directions of motion in a random dot display and to report the perceived direction with an eye movement (saccade) to one of two visual targets. In each trial the subject is presented with the visual stimulus of dots drifting left or right across the screen for a fixed duration. Once the dots disappear, and after a delay, a “go” cue is given to prompt subjects to indicate their decision about the direction in which the dots are moving—by looking either to the left or right—with a mean reaction time of 245 ms.

We use data from a well-known experimental paradigm, the Random Dot Motion discrimination task (RDM) (Shadlen and Newsome, 2001; Kiani and Shadlen, 2009; Kiani et al., 2014, 2015), in which the subject must decide which direction dots on a screen are moving (task described in Figure 1). The “coherent output” in this experiment is the decision. To study the computation of the output, we analyze the activity of 169 neural channels in a macaque monkey performing the task. The recorded neurons are located in the prearcuate gyrus in prefrontal cortex (area 8Ar) (Kiani et al., 2015). Area 8Ar has been implicated in motor planning and control of eye movements as described below. The recording is achieved using a multi-electrode array of size 4 mm × 4 mm (see **Figure 10**). Spikes are sorted using standard techniques, mapping spikes detected by each electrode onto a set of unique neural units, each of which represent the activity of one or a few individual neurons (Kiani et al., 2015).

The measured neural activity is qualitatively different before and after the go cue, demarcating two time intervals that we call Phase I and Phase II (Figure 1).

### 2.1. Previous work

The causal pathway for perceptual decisions in the primate brain is still debated. The lateral intraparietal cortex (LIP) has been a contender as a causal decision-making locus, as it demonstrates accumulation of perceptual evidence (Shadlen and Newsome, 2001; Huk and Shadlen, 2005; Gold and Shadlen, 2007; Kiani and Shadlen, 2009; Hanks et al., 2014) and because stimulating LIP neurons can lead to more quickly reaching a decision threshold (Hanks et al., 2006). Yet a recent study has shown that inactivating large sections of LIP has little effect on decision-making (Katz et al., 2016), suggesting that other areas with which LIP is closely associated may redundantly play a causal role (Hanks and Summerfield, 2017). Such redundancy is also suggested by a study in analogous brain regions in rats (Erlich et al., 2015).

Here, we use data from one of these closely related areas, area 8Ar in dorsolateral prefrontal cortex. Area 8Ar, like LIP, carries information about planned saccades in direction discrimination tasks (Kim and Shadlen, 1999; Constantinidis and Goldman-Rakic, 2002; Hussar and Pasternak, 2009; Kiani et al., 2014, 2015). The accuracy, latency, and confidence in the decision are influenced by noise in the input (experimentally controlled in the RDM task by introducing random motion in the stimulus that varies the percentage of dots moving coherently in one direction), which has measurable effects in both prefrontal cortex (Kim and Shadlen, 1999) and LIP (Shadlen and Newsome, 2001). This sensitivity to the strength of the input suggests that these brain areas do not only represent the decision once made, but are also involved in the decision making process, accumulating information about sensory input.

At the most abstract level, decision-making can be fitted using a variety of continuous or discrete one-dimensional random walks or diffusion models with fixed or variable thresholds (Gold and Shadlen, 2007). A number of closely related simple neural network models, extended to include lateral inhibition or recurrent activation, recover several features of these empirical findings (Gold and Shadlen, 2007; Ratcliff and McKoon, 2008), including timescales in the decision process that are much slower than those of individual neurons. More detailed mechanistic models have successfully reproduced important aspects of the observed decision-making process using individual spiking neurons and also emphasize the emergence of slowly acquired information at the neuronal level using the terminology of recurrent “reverberation” (Wang, 2002). (See Discussion for more details about existing models.)

**Figure 2:**
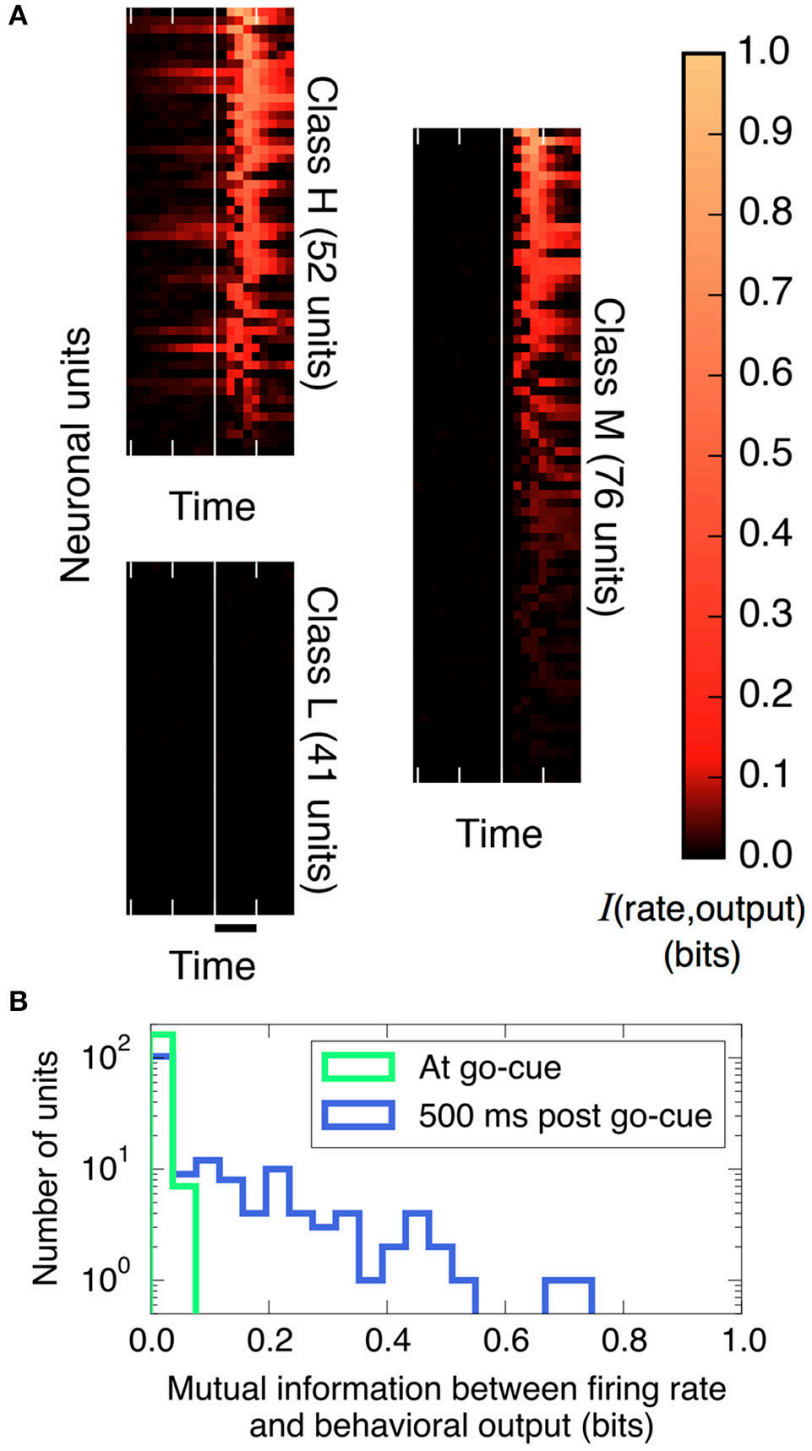
The amount of information that individual neural units encode about the decision output varies strongly over units and over trial time. **(A)** Plotted is the mutual information between spike rates of individual neural units and the decision (using Equation 9, with 200 ms bins), aligned by the go cue at the vertical white line (time scale bar corresponds to 500 ms). Before the go cue, only a few units predict the decision, but afterward, many do. Basis for classifying neurons: those in Class H encode information before the go cue, those in Class M encode information after but not before the go cue, and those in Class L never encode information. (We group in this way to conveniently display the diversity of the behavior of individual neural units; these groups are not meant to indicate statistically distinct clusters.) **(B)** Histogram of information encoded by each unit at and after the go cue. At the go cue (light green), no individual units encode more than 0.1 bits about the decision, but half a second later (dark blue), many do.

Most of these models assume a single phase in which information is accumulated. They do not, however, explicitly consider the collective properties of this accumulation—is the information about the decision localized in individual neurons or encoded at the population level? Furthermore, is accumulated information shared or transmitted across the population of neurons? The observation that neuronal behavior is qualitatively different before and after the go cue (see Figures 1, 2) minimally suggests that there are two processes taking place, rather than just one accumulation phase.

Existing decision-making literature tends to neglect neural behavior after the go cue, treating it as “choice execution.” We argue that post-go-cue behavior is an extension of decision making at the system level and view the process between the go cue and the saccade as essential to collective decision-making—“reading out” the information that is, before this point, only available by pooling information from many cells.

## 3. Proposal and summary of results

In this paper we propose, building on previous work (Flack, 2012; Flack et al., 2013), that collective computing systems are characterized by two phases—slow aggregation and fast propagation. The idea is that this two phase computation is useful when the system has many imperfect sensors each forming an opinion based on incoming data. In the case of the study system, in order to both accumulate information about a temporally extended signal and retain it during the delay period, we expect that individual cells should form collectives that can accumulate information over hundreds of milliseconds by (1) sharing information through recurrent excitation but (2) avoiding committing to a decision too early.

Hence in Phase I (slow aggregation) we propose information is acquired through a process of sensory accumulation. To improve the reliability of the information given noisy input and propensity for error at the component level, a sum or other integration is performed at the population or subpopulation level. This is essentially crowd-sourcing. In the measured neurons, this happens during the stimulus presentation and delay period (Figure 1).

In Phase II (fast propagation) we propose that information at the level of units in Phase I is propagated quickly across a population of cells that may or may not have participated in Phase I. The outcome of propagation is neural consensus in so far as it results in the decision being encoded in each individual neuron. This consensus allows the system to act.

In our study system we find evidence for both Phase I and Phase II. Our results suggest Phase II occurs post-go-cue and is achieved through increased information amplification and sharing.

Finally, we develop a dynamical rate model that explains this behavior in terms of varying distance from a symmetry-breaking transition. In the simplest form of the model, this distance is controlled using a time-varying recurrent excitation among informative neurons. The model demonstrates a fundamental connection between timescales and redundancy, with the formation of a collective slow timescale requiring a population with lower informational redundancy.

## 4. Results

### 4.1. Heterogeneity of individual information over space and time

First, we quantify how much information about the decision is encoded in individual neural firing rates. We find substantial heterogeneity over neurons and as a function of time. As shown in Figure 2A and described in the figure legend, we can group neurons into three classes based on when their firing patterns encode information about the decision.

Figure 2B compares histograms of mutual information at the time of the go cue and 500 ms later, demonstrating that while no individual neuron encodes more than 0.1 bits of information about the decision at the time of the go cue, many individual neurons encode a substantial amount of information during and after the saccade indicating the decision. This observation motivates splitting the process into two distinct temporal periods, Phase I (pre go cue) and Phase II (post-go cue) (Figure 1).

### 4.2. Switching of collective information from synergistic to redundant

We next assess whether information about the decision is encoded collectively at the whole population level or within a subpopulation and how this quantity compares to the information encoded at the individual neuron level.

**Figure 3:**
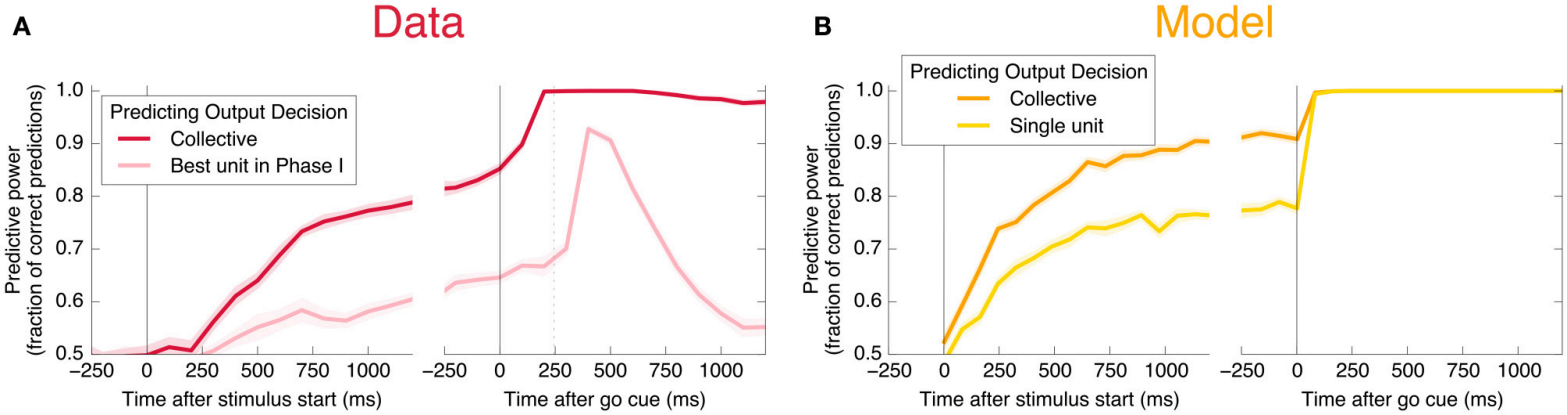
Neurons encode information about the output decision, with distinct dynamics in Phase I (pre-go cue) and Phase II (post-go cue). **(A)** Predictive power with respect to the output decision, for the single most informative unit in Phase I (light color) and, using LDA, for the entire population (dark color). Information about the decision grows slowly in Phase I and jumps suddenly in Phase II. The jump happens before the mean time of the saccade, indicated by the vertical dotted line. (Note that data after the saccade is likely to be influenced by visual feedback due to the motion of the eye.) **(B)** This qualitative behavior is reproduced by a simple rate model in which the degree of recurrent excitation is increased in Phase II. Corresponding mutual informations are plotted in **Figure 11**.

An encoding based on Linear Discriminant Analysis (LDA) allows us to verify that the population encodes more information than any single neural unit by producing a lower bound on the mutual information encoded jointly by the entire population (see Methods). As shown in Figure 3A, this collective encoding is able at the time of the go cue to predict the decision on more than 80% of out-of-sample trials, corresponding to a collective mutual information (CMI) reaching about 0.5 bits (**Figure 11A**). Shortly after the go cue, CMI rises to nearly 1 bit, with nearly perfect out-of-sample predictions.

**Figure 4:**
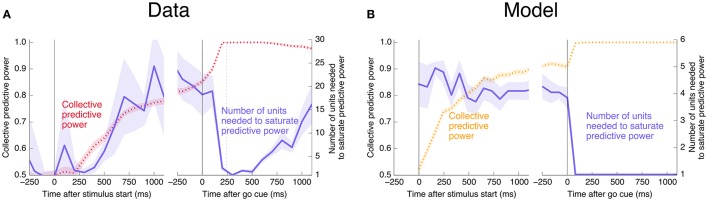
At the time of the physical implementation of the decision, encoding shifts: Phase I resembles a population code, whereas in Phase II individual rates are predictive. **(A)** The number of cells required to be measured in order to saturate in predictive power is plotted in purple, defined as the number needed to reach performance relative to chance in an LDA encoding that is 95% of performance using all 169 units (red, same as plotted in Figure 3). Up to the time of the go cue, most neurons individually encode little information about the output (see also Figure 2), but are highly predictive when the activity of many neurons is combined. Predictive power saturates only once about 20 neurons have been measured. Shortly after the go cue, and just before the typical time of the saccade (dotted line), the population reaches perfect predictive power, while at the same time individual neurons become highly predictive—maximal information can be obtained by measuring only one to two neurons. See Figure 13 for an analogous plot aligned to the saccade. **(B)** The simple rate model reproduces these qualitative features.

Interestingly, at the same time that the collective information jumps to its maximum value, there is a switch in the distributed nature of the encoding: many individual units become highly informative in Phase II, providing redundant information. This contrasts with Phase I, in which much more information is contained at the population level than in any single unit. To quantify the redundancy of the encoding, we ask how many units need to be included in the LDA encoding in order to reach 95% of the maximal collective predictive power. As shown in Figure 4, about 20 units need to be included for peak performance in Phase I, but this drops sharply to 1 or 2 at the time of the saccade. (See **Figure 13** for a version of this plot aligned by saccade time, showing explicitly that this decrease in distributedness starts before the saccade.)

**Figure 5:**
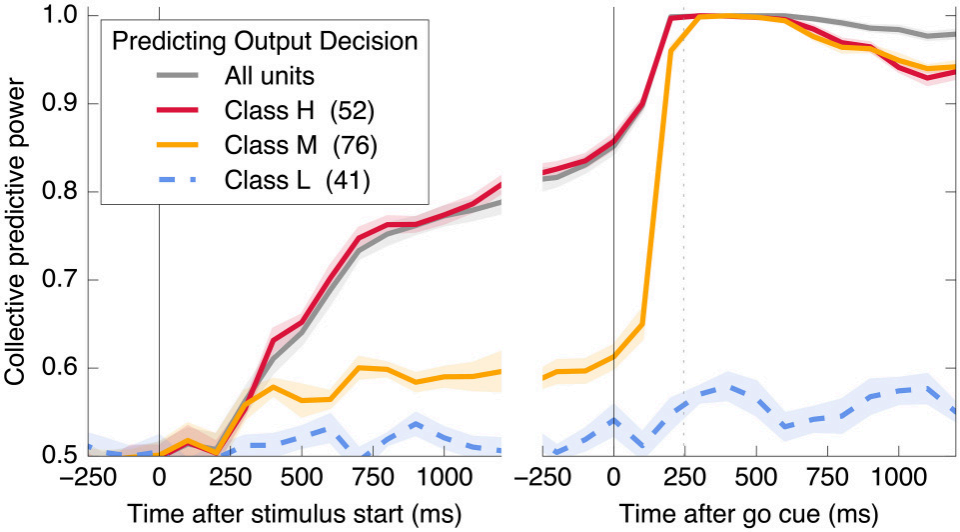
A subset of neurons collectively encodes information about the decision in Phase I, and more neurons contain information in Phase II. Information about the decision gradually builds during Phase I in some neurons (Class H; red), whereas in other neurons (Class M; orange), little information is present until just before the saccade representing the decision (mean saccade time indicated by dotted line). Class L units (blue) always contain little information.

Figure 5 demonstrates that all information about the decision is encoded in class H units in Phase I, and that this changes to include class M units in Phase II. In addition, information is acquired over a longer timescale in Phase I than in Phase II. Table 1 summarizes the observed properties of the three classes.

**Table 1:** Qualitative summary of informational properties of each neural class.

**Neural class**	**Phase I information**	**Phase II information**
H	Synergistic, slow growth	Redundant, fast growth
M	Uninformative	Redundant, fast growth
L	Uninformative	Uninformative

**Figure 6:**
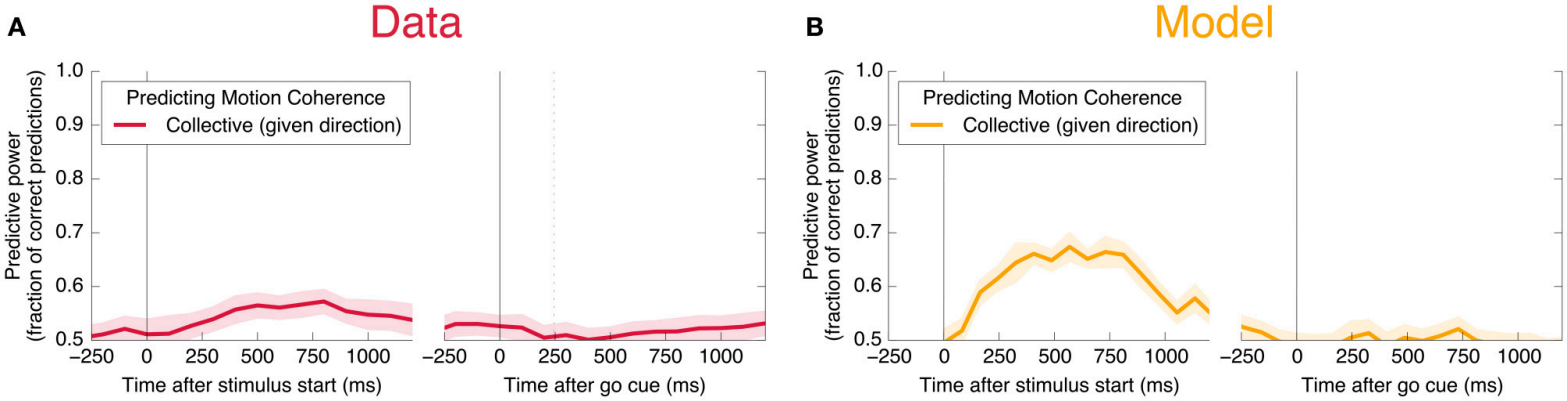
Neurons encode information about the input stimulus, but much less than about the output decision. Analogous plots to Figure 3 for predicting the coherence of the visual stimulus. Information about the coherence is small but visible in Phase I, and disappears in Phase II. Single unit traces are not included on these plots because they are not significantly different from zero. Corresponding mutual informations are plotted in Figure 11.

We also find information at the population level specifically about the input stimulus, but it is small compared to the information about the decision, and is significant only during Phase I. This can be seen in **Figure 11C**: at its peak during Phase I, we estimate that the LDA encoding provides only about 0.02 bits of information per trial about whether the coherence of the input is strong or weak (out of a possible 1.0 bit; see Appendix). Though this value is similar to the uncertainty in our estimate of the mutual information, we can be confident that it is nonzero by noting that the LDA encoding can predict the coherence of out-of-sample trials significantly better than chance (Figure 6A).

### 4.3. Information accumulation and consensus formation using a dynamic rate model

We explore the relationship between timescales of information accumulation, memory, and informational redundancy using a simple dynamical model. We start simply by representing individual neurons as having a state that (1) is persistent on the timescale of tens of ms, (2) transiently affects the states of other neurons via a firing rate that saturates as a function of the current state, and (3) is subject to random noise. The model consists of *N* homogeneous, all-to-all coupled neurons with individual firing rates *r*_*i*_ = tanh*x*_*i*_, whose dynamics are governed by:

(1) $$\begin{array}{l} \tau \frac{dx_{i}}{dt}=s-x_{i}+\xi +c\underset{j\neq i}{\sum }r_{j}. \end{array}$$

Here, *s* represents an input signal with magnitude proportional to the coherence of the visual stimulus and sign indicating the dominant direction of motion, *c* is the strength of positive interactions between every pair of neurons, τ = 10 ms sets the timescale of decay for a single neuron, and the final term represents noise with variance Γ^2^ [drawn from a Gaussian distribution for simplicity: $\langle \xi \left(t_{0}\right)\xi \left(t_{1}\right)\rangle =\delta \left(t_{1}-t_{0}\right)N\left(0,\Gamma^{2}\right)$]. See Methods and Appendix for additional details motivating this form for the dynamics. We assume that the *N* neurons are fully responsible for making the decision, and that the experiment measures some subset of these units (*N*_measured_ < *N*). As shown in Figures 3, 4, 6, **8**, (see also **Figures 11**, **12**) we find that this model can capture both the integration and storage of the decision during Phase I and the consensus formation and propagation of the decision during Phase II.

**Figure 7:**
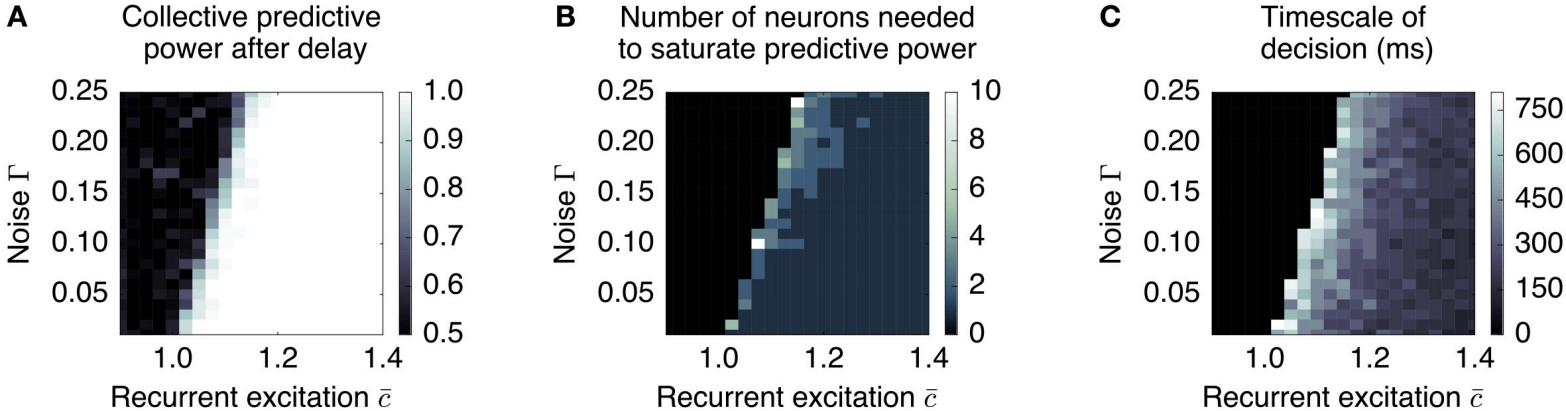
Long timescales and low redundancy occur near the collective transition at which memory storage becomes feasible. We plot three aspects of the collective behavior produced by a simple distributed, dynamic rate model as a function of recurrent excitation and noise, for *N* = 500. **(A)** The degree to which the system can retain a stable memory is measured by the proportion of times one can be successful in predicting the sign of the mean pre-delay state given the post-delay state. This delineates the transition between monostable and bistable systems. **(B)** The system is less redundant near the transition, requiring more neurons to be measured to reconstruct the memory. **(C)** The timescale over which certainty in the eventual choice builds (measured as the first time at which the predictive power reaches 95% of its final value) is largest near the transition. Regions that do not successfully retain a memory (performance less than 0.9) are blacked out in plots **(B,C)**.

Our simple dynamic rate model model produces behavior that is critically dependent on the degree of recurrent excitation, controlled by $\bar{c}=c\left(N-1\right)$, and the amount of neural noise Γ (Figure 7). For fixed Γ, there exists a critical value of recurrent excitation ${\bar{c}}^{*}$ such that for $\bar{c}<{\bar{c}}^{*}$, the only stable attractor is at $\vec{r}={\vec{r}}_{0}=\vec{0}$, while for $\bar{c}>{\bar{c}}^{*}$, two stable attractors, ${\vec{r}}_{+}$ and ${\vec{r}}_{-}$, emerge symmetrically on two sides of ${\vec{r}}_{0}$ (see Appendix).^1^ Bistability is required for persistent activity that remembers the decision during the delay period. The informative direction in rate space lies along the vector $\vec{v}={\vec{r}}_{+}-{\vec{r}}_{-}$, which in this simple model weights all units equally and with the same sign.

The degree of recurrent excitation $\bar{c}$ also controls two other aspects of decision-making: (1) the timescale τ_decision_ over which information is accumulated and (2) the redundancy of the encoding of the decision.

First, the relevant timescale for motion along the decision direction $\vec{v}$ is inversely related to the distance from the instability threshold ${\bar{c}}^{*}$ (see Figure 7C): e.g., without noise (Γ = 0), ${\bar{c}}^{*}=1$, and $\tau_{\text{decision}}=\tau /\left(\bar{c}-{\bar{c}}^{*}\right)$. This is the phenomenon of “critical slowing down,” which slows motion along $\vec{v}$ when the system is near the threshold.^2^ Hence, units that forget on a short timescale can still contribute to aggregate-level behavior that integrates over longer timescales. This emergent timescale has been recognized in “reverberation” models (Wang, 2002) as an essential feature of distributed decision-making.

Secondly, sufficiently close to the threshold, noisy individual cells are only weakly constrained to have a similar state as the others; whereas summing over many cells can reliably predict the behavior of the whole, individual cells do not encode much information. This corresponds to the “synergistic” state of Phase I. As recurrent excitation increases, consensus is more strongly enforced, leading to individuals containing more information. This corresponds to the “redundant” state of Phase II. This dependence is demonstrated in Figure 7B: the number of units one needs to measure to obtain maximal information is largest near the transition point.

**Figure 8:**
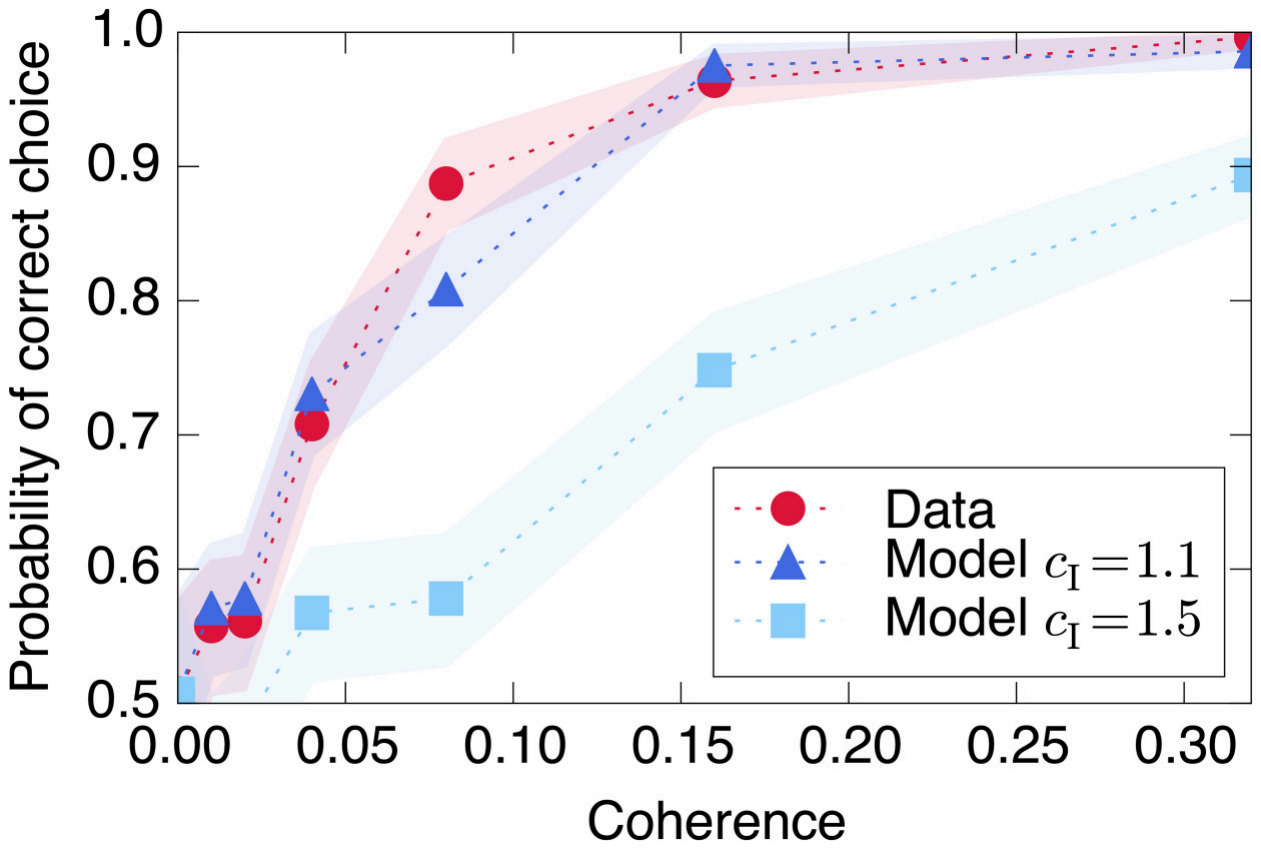
Decision-making model performance decreases if recurrent excitation is large throughout the entire trial. The strength of the model's input signal *s* is calibrated with a single parameter such that it reproduces the empirical performance curve (red circles) using relatively small recurrent excitation ${\bar{c}}_{I}=1.1$ (dark blue triangles). If the model is modified to have the same $\bar{c}$ through both Phase I and Phase II (light blue squares), the decision is made too quickly and performance decreases at moderately difficult coherence levels. Shaded regions represent bootstrapped 90% confidence intervals.

**Figure 9:**
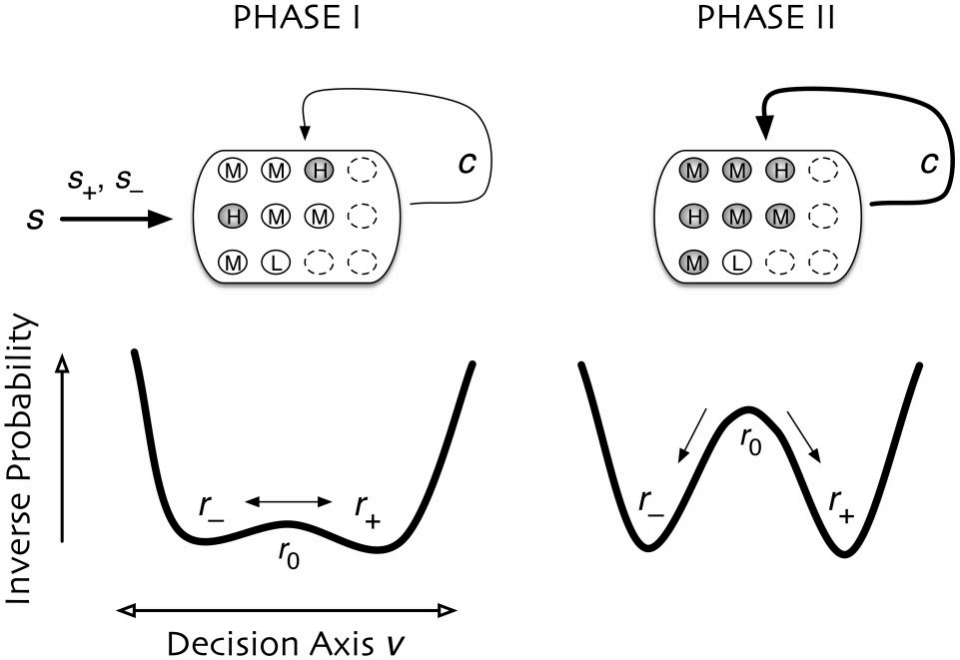
Dual coding through critical slowing down. During Phase I the state of cells is determined largely by the coherence of the input signal, *s*. A small population of cells *H* are able to slowly accumulate information (filled circles) about the input *s*. Cells of type *M* and type *L* accumulate no information (open circles). In Phase II the informational state of cells is dominated by recurrent excitation *c* among the cells. Cells of type *H* and *M* become highly informed whereas cells of type *L* remain uninformed. In Phase I, the system is governed by stochastic dynamics with a timescale set by “critical slowing down,” in which the system in the initial state ${\vec{r}}_{0}$ slowly moves between two decision states ${\vec{r}}_{+}$ and ${\vec{r}}_{-}$. The symmetry is weakly broken by the coherence of the input signal, slowly biasing the system into one state. In Phase II the symmetry is strongly broken and one of two decision states is rapidly approached. Accuracy is determined in Phase I whereas consensus is reached in Phase II.

The combination of low redundancy and slow dynamics therefore suggests that Phase I should correspond to $\bar{c}$ only slightly greater than ${\bar{c}}^{*}$, and large redundancy in Phase II suggests a larger $\bar{c}$. Indeed, as displayed in Figures 3, 4, 8, the simple rate model reproduces the qualitative behavior of the system by changing the single parameter $\bar{c}$ between Phase I and II, with ${\bar{c}}_{II}>{\bar{c}}_{I}>{\bar{c}}^{*}$. Specifically, the increased redundancy of Phase II is associated with a faster timescale. Together, these results imply a dual coding theory for collective decision-making through critical slowing down, summarized in Figure 9.

Additionally, the model explains how neurons responsible for the decision can encode a relatively small amount of information about the input stimulus coherence. As the attractors representing the decision do not depend on the coherence, information is only contained in the speed with which those attractors are approached. This speed is in turn related to the magnitude of the input current (*s*), which can be small if *N* is sufficiently large to magnify a small signal.

Using a simple linear relationship between stimulus coherence and input *s* produces a good fit to the observed psychometric function, shown in Figure 8. The figure also demonstrates the model's prediction (as in Wang, 2002) that using the same large recurrent excitation $\bar{c}$ in both Phase I and Phase II would lead to a faster decision process, which would integrate the input stimulus over a shorter time and therefore produce poorer performance in trials with more ambiguous, less coherent visual stimuli.

## 5. Discussion

In this study we used information theory and the theory of collective phenomena to analyze time series data from a microelectrode array capturing 169 neural channels in the prefrontal cortex area 8Ar of a macaque monkey. This is a well studied area of the brain that has been shown to play an important role in both visual decision-making and motor behavior (Kim and Shadlen, 1999; Constantinidis and Goldman-Rakic, 2002; Hussar and Pasternak, 2009; Kiani et al., 2014, 2015). Our findings lead us to propose a coding-duality framework, applicable to collective computation in adaptive systems more generally, that includes a slow accumulation process in which information is encoded in populations and a fast consensus formation process in which information is encoded redundantly in multiple individual neurons.

In the neural time series studied here, the idea of Phase I as information accumulation is in good agreement with many prior studies of MT, LIP, and prefrontal cortex in which information is integrated at the population level (essentially through crowd-sourcing) in order to increase the accuracy of a decision (Kiani et al., 2008, 2014; Kiani and Shadlen, 2009; Fetsch et al., 2014) and reduce decision latency (Huk and Shadlen, 2005; Hanks et al., 2014; Kira et al., 2015).

Neural behavior post-Phase I has received less attention. Our results suggest a second phase, during which a large subset of cells becomes correlated and acquires redundant information extremely rapidly. This phase of “consensus formation,” in which information rapidly spreads from the “knowledgeable” neurons to many neurons, dramatically increases redundancy in the system. Our simple rate model accomplishes this switch by changing the degree of recurrent excitation, but it could alternatively be controlled by external inputs to the circuit through a perturbation that moves the system away from the symmetry-breaking transition.

Our results suggest investigating other forms of neural decision making to look for similar dynamic consensus phenomena. We expect the separation of timescales between Phases I and II to be most clear in cases involving a gradual accumulation of evidence, such as comparing two extended auditory signals (Erlich et al., 2015). In vibrotactile decision making, a similar mechanism has already been proposed for carrying out multiple phases of a decision process using a single population of neurons (Machens et al., 2005), which suggests looking for comparable patterns of changing consensus.

Although there has been little focus on Phase II and, more generally, consensus formation, in neuroscience, the role of consensus formation in collective computation has been a focus in the study of social processes. For example, search engines and auctions illustrate both slow accumulation and fast consensus (e.g., Leise, 2006; Brush et al., 2013, see also Allesina and Pascual, 2009 for related results for food webs). In order to generate an approximate importance or price for a website or good, information needs to be acquired from a variety of independent sources through search trends or bids. At this stage (accumulation of individual decisions), it is best for sources not to interact, to avoid premature consensus before all relevant information can be gathered. Secondary web sites and sellers can then very quickly establish new prices and strategies by polling the sale price or popularity of comparable products from those that have already accumulated this information. This generates increased correlations among both markets and web sites (consensus on a collective decision).

Collective computation based on information accumulation and consensus formation has also been observed in the formation of power structures in primate societies (Flack and Krakauer, 2006; Flack, 2012; Brush et al., 2013; Brush, in press). Individual monkeys acquire information about the ability of other monkeys in the group to use force successfully during fights, and they communicate this slowly changing perception using special status signals (Flack and de Waal, 2007)—signal emission is the analog to neural firing or linking web pages. The degree of consensus or agreement in the group about any single individual's ability to use force is its “social power” (Flack and Krakauer, 2006). Consensus about power is encoded in the network of status signaling interactions just as consensus about value in the search engine example is encoded in links making up the World Wide Web. It has been shown in prior work that the same algorithms can be used in both the search engine and power cases to quickly and efficiently calculate the consensus encoded in the networks (Brush et al., 2013).

In all three examples (neural, search, power) accumulation is slow and consensus is fast. In the power example it has additionally been shown that an advantage of this timescale separation in collective computation is that it produces a slowly changing yet accurate power structure that serves as a reliable “background” against which individuals can, on a fast timescale, tune strategies quickly and effectively (Flack, 2012; Flack et al., 2013). This is also likely to be true for the search engine case but it remains unclear how this kind of ‘timescale separation dependent feedback’ could play a role in the neural case.

One additional important difference between the neural case and the social cases is that in the social cases both accumulation and consensus can be occurring simultaneously but on different timescales. In the neural case presented here, accumulation (Phase I) occurs first with consensus (Phase II) following, but this may be an artifact of the experimental setup with an externally forced go-cue.

In large systems that are processing information from multiple sources it is difficult to conceive of any way of achieving an efficient, accurate, coordinated representation of environmental regularities other than through a dual-process dynamic. This is because (1) it takes time to integrate information from noisy sources, and (2) not all cells have equal access to information and therefore must acquire input from informed cells. We refer to this requirement as “coding-duality” as it implies a shift from an emphasis on populations of cells pooling resources in Phase I to single cells in possession of all adaptive information through consensus mechanisms in Phase II.

These results help clarify the debate between proponents of the modern neuron-doctrine and distributed-representation theory (Bowers, 2009; Quian Quiroga and Kreiman, 2010). In the data-set we have analyzed, both processes are occurring but at different temporal phases of the decision task. By restricting analysis to only one phase, or averaging over time, the ability to resolve the bi-phasic distinction is lost and one or the other extreme—informed single cells or informed populations of cells—is statistically favored.

### 5.1. Area 8Ar neurons primarily represent the motor decision, yet could be solely responsible for mapping sensory information to a decision

In prior studies the representational status of areas 8Ar and LIP has remained ambiguous, and is often described as partly sensory and partly motor. We find that whereas spiking activity in 8Ar cells is strongly predictive of saccadic eye-movements, there is little residual information concerning the visual stimuli (Figures 3, **11**). In other words, these cells are primarily predictive of motor behavior and not sensory input, and in this informational sense are almost purely motor.

Yet this does not rule out the measured neurons being part of a group of similar cells that are collectively fully responsible for the decision. In the rate model, the simulated cells are fully responsible for the decision but measuring a subset of the cells reveals only a small amount of information about the input signal—and even this information is quickly lost once the system reaches an attractor state representing the decision. Thus, because we have data on only a small fraction of all neurons in these areas, it is feasible that 8Ar neurons as a whole could be solely responsible for mapping sensory data onto the decision.

### 5.2. Relationship to known classes of neurons

Many previous studies that attempt to model the perceptual decision-making system (e.g., Wang, 2002) and neural computation more generally (see Lisman, 2015 for a review) have focused on interactions within and between two distinct groups of neurons: the pyramidal neurons and the inhibitory interneurons.

These neural classes are typically identified from electrode data by differences in firing rate, spiking waveform, burstiness, and refractory period (e.g., Csicsvari et al., 1999; Diba et al., 2014). Pyramidal cells are typically more informative about (selective with respect to) sensory input than interneurons (e.g., Diba et al., 2014). Additionally, sensory selective cells and/or cells identified as pyramidal have been shown in many contexts to fire more independently of one another: pyramidal compared to parvalbumin-expressing interneurons in the visual cortex (Hofer et al., 2011); visually responsive neurons in V1 (Ecker et al., 2010); excitatory vs. inhibitory neurons in the prefrontal cortex (Constantinidis and Goldman-Rakic, 2002); pyramidal vs. interneurons in rat hippocampus (Diba et al., 2014). This suggests classifying our Class H (and perhaps Class M) units as pyramidal cells and Class L as interneurons.

### 5.3. Relationship to other models of decision-making

In Table 2, we compare the model of Equation (1) to related binary decision-making models that vary in their level of detail. The equations shown here use a single population, but an equivalent formulation can be found in each case consisting of two competing populations (Usher and McClelland, 2001; Gold and Shadlen, 2007). We do not include models using discrete neural firing rate states (e.g., Latimer et al., 2015), which we expect to have equivalent behavior at the collective level.

**Table 2:** Related perceptual binary decision-making models.

**Model**		**Input**	**Noise**	**Decay**	**Feedback**	**Decision mechanism**	**Equilibria**
Drift diffusion (Bogacz et al., 2006)	$\frac{dx}{dt}$ =	*s*	+ξ			Threshold	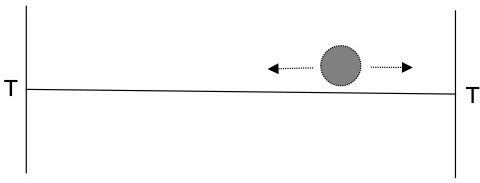
Leaky integrator (Kiani et al., 2008)	$\frac{dx}{dt}$ =	*s*	+ξ	−*x*		Threshold	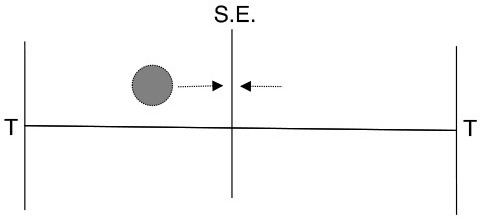
Ornstein-Uhlenbeck (OU) (Usher and McClelland, 2001)	$\frac{dx}{dt}$ =	*s*	+ξ	−*x*	+*cx*	Threshold	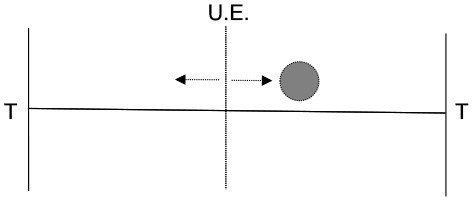
Distributed saturating OU [Equation (1)]	$\frac{dx_{i}}{dt}$ =	*s*	+ξ	−*x*_*i*_	$+c\underset{j}{\sum }r\left(x_{j}\right)$	Attractor	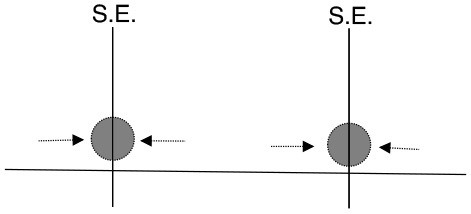
Distributed spiking models (Wang, 2002)	Detailed spiking dynamics					Attractor	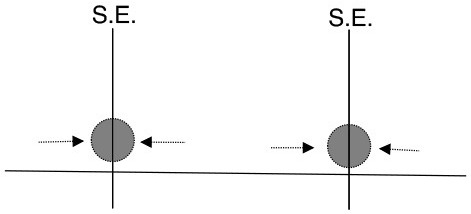

*In the Equilibria column, we schematically represent equilibria along the dimension of the decision variable, assuming c > 1. T, threshold; S.E., stable equilibrium; U.E., unstable equilibrium*.

The simplest models are most analytically tractable, abstracting away both the distributed nature of the computation and mechanisms for saturation and persistent memory, beginning with the simplest drift diffusion model (Bogacz et al., 2006). These models assume that a separate mechanism makes and stores the decision once a stochastic process reaches a given threshold. This simple process successfully explains the integration of sensory information, the distribution of reaction times, and accuracy as a function of input coherence.

However, models that do not include decay or saturation of firing rates cannot explain the loss of information about the coherence of the input late in the trial (Figure 6A). Diffusive models constrained to stay within specified bounds could potentially alleviate this problem, as could so-called ‘leaky integrators’ with a state that decays back to a starting value (Kiani et al., 2008). Such models have been ruled out because they predict persistent susceptibility to sensory evidence presented late in the trial, which is not seen in experiments (Kiani et al., 2008).

Instead, the loss of information about coherence and the loss of susceptibility can be parsimoniously explained if the decision process involves approaching stable attractors that store the decision during the delay period. Including feedback is one way to produce multiple attractors. In the simplest case, this leads to the Ornstein-Uhlenbeck (OU) model (Busemeyer and Townsend, 1993; Bogacz et al., 2006), which produces multiple attractors when combining positive feedback with saturation or boundedness (Zhang and Bogacz, 2010).

None of these low-dimensional models, however, address the issue of how the computation is distributed over multiple cells. The noisiness of individual neurons likely necessitates larger populations of neurons in order to accumulate persistent information over longer timescales.

Existing models that incorporate collections of cells include those with populations of spiking neurons (Wang, 2002; Lo and Wang, 2006; Wimmer et al., 2015) or of neurons with continuous states governed by noisy dynamics (Wong and Wang, 2006). As in our model, these studies have emphasized the importance of recurrent interactions to slow the effective timescale using the phenomenon of “reverberation,” essentially equivalent to critical slowing down. One proposed model also includes separate neurons that act as a switch governing the transition from information accumulation to decision commitment (Lo and Wang, 2006). This is distinct from the single-population story we present here, instead hypothesizing that information accumulation and consensus happen in different brain regions. Generally, these studies have not explicitly addressed the distributedness of neural information, but we expect the models may display similar phenomena with respect to population dynamics, coding, and redundancy. It will be useful in future work to confirm this.

The model presented in Equation (1) captures the details necessary to describe the distributed nature of the computation but abstracts away most details of neurobiology. It can be viewed as a distributed implementation of an Ornstein-Uhlenbeck model (Busemeyer and Townsend, 1993; Bogacz et al., 2006; Zhang and Bogacz, 2010) that includes biologically realistic saturation of firing rates.

### 5.4. Outlook

The results we find here in the specific case of the random dots task in prefrontal cortex hint at more general design principles for decision-making and collective computation. For instance, much attention has been paid to the idea that it may be beneficial for collective information processing systems to exist near a symmetry breaking transition, or critical point (Langton, 1990; Mora and Bialek, 2011; Plenz and Niebur, 2014). We can come to a similar but more subtle conclusion in our dynamic model: a system near a transition (our Phase I) is indeed successful in producing distributed, collective states that remain more sensitive to exogenous inputs (as has been emphasized in “criticality” research; e.g., Shew et al., 2009), but reaching consensus (in Phase II) requires moving away from the transition toward a collectively boring but useful “frozen” state. Locating, managing, and controlling dynamics with respect to such transitions and collective states is certainly important for not only a brain involved in cognition and learning, but in controlling collective behavior across many biological and social systems.

## 6. Methods

### 6.1. Measuring association: mutual information

Mutual information is the information shared by any two streams of data *A* and *B*. This can be thought of in information theoretic terms as the average number of bits that are revealed about *B* upon measuring *A*. For instance, with *A* representing a firing rate and *B* the behavioral output (a choice of either left or right), the mutual information *I*(*A, B*) represents how much information the neuron's rate provides about the decision variable. The mutual information between *A* and *B* is the sum of the marginal entropies (the maximum potential information content of the paired system) minus the joint entropy (the amount of noise in the paired system); see Appendix.

In these analyses, we employ rates of neural firing averaged over time bins of length 200 ms (assuming rate encoding and ignoring details about the precise timing of spikes) in order to calculate dependencies among neural and behavioral states using mutual information. Entropies are estimated using the NSB method (Nemenman et al., 2004, 2002). We consider a mutual information to be significant if it is greater than 0.01 bits, roughly the resolution of entropy estimation for the amount of data we have, which is also estimated using the NSB method.

### 6.2. Linear discriminant analysis for population encoding

To combine neural firing rates into a collective encoding, we use LDA. Given neural rate data from many trials and classification of those trials as left decisions and right decisions, LDA attempts to find the linear combination of neural rates that is most informative of the class (left or right).

LDA makes the simplifying assumption that data from each class is produced by a multidimensional Gaussian specified by the observed mean ${\vec{\mu }}_{\alpha }$ and covariance matrix *C*_α_ for each class α. In this case, projecting any given data vector $\vec{r}$ along the LDA vector, defined as

(2) $$\begin{array}{l} \vec{v}={\left(C_{1}+C_{2}\right)}^{-1}\cdot \left({\vec{\mu }}_{2}-{\vec{\mu }}_{1}\right), \end{array}$$

produces a number designed to be informative about the class from which the data vector came (with maximal performance guaranteed when *C*_1_ = *C*_2_). That is, the LDA vector $\vec{v}$ provides a weighting of individual neural rates that, when summed, approximately maximally separates the two classes.

LDA simultaneously provides a framework for predicting the output given rate data. Looking at any given set of rates $\vec{r}$, the LDA's estimate of the relative log-likelihood of left vs. right can be written as:

(3) $$\begin{array}{l} L\left(\vec{r}\right)=\log\left(\frac{\text{likelihood }\vec{r}\text{ came from left trial}}{\text{likelihood }\vec{r}\text{ came from right trial}}\right) \end{array}$$

(4) $$\begin{array}{l} =-\frac{1}{2}\log\left(\vec{v}\cdot C_{1}\cdot \vec{v}\right)-\frac{{\left(\left(\vec{r}-{\vec{\mu }}_{1}\right)\cdot \vec{v}\right)}^{2}}{2\;\vec{v}\cdot C_{1}\cdot \vec{v}}\\ +\frac{1}{2}\log\left(\vec{v}\cdot C_{2}\cdot \vec{v}\right)+\frac{{\left(\left(\vec{r}-{\vec{\mu }}_{2}\right)\cdot \vec{v}\right)}^{2}}{2\;\vec{v}\cdot C_{2}\cdot \vec{v}}. \end{array}$$

Thus, positive $L\left(\vec{r}\right)$ corresponds to a (maximum likelihood) prediction that $\vec{r}$ came from a left trial, and negative to prediction of a right trial.

### 6.3. Dynamic, stochastic, distributed decision-making model

Equation (1) describes a dynamic Hopfield network (Hopfield, 1984) that includes Gaussian noise on neuron states and is restricted to uniform positive interactions among all cells.

We imagine that we measure some subset *N*_measured_ of *N* neurons that are collectively responsible for the decision, with no input from other areas of the brain except for a temporary signal *s*, which is proportional to the signed coherence of the visual stimulus.

Although this simplified picture does not include spiking, models that include spiking have been shown to produce similar behavior (Wang, 2002). Our model can be mapped onto a model with separate populations that are positively selective for leftward and rightward stimuli by assuming that (1) the variables *r*_*i*_ represent the difference from a typical firing rate, (2) oppositely selective cells are mapped onto *r*_*i*_ with opposite sign while simultaneously changing the sign of *s*_*i*_ and the sign of *c*_*ij*_ when *i* and *j* are oppositely selective cells, corresponding to mutual inhibition between opposite populations.

## 7. Appendix

### 7.1. Experimental data

**Figure 10:**
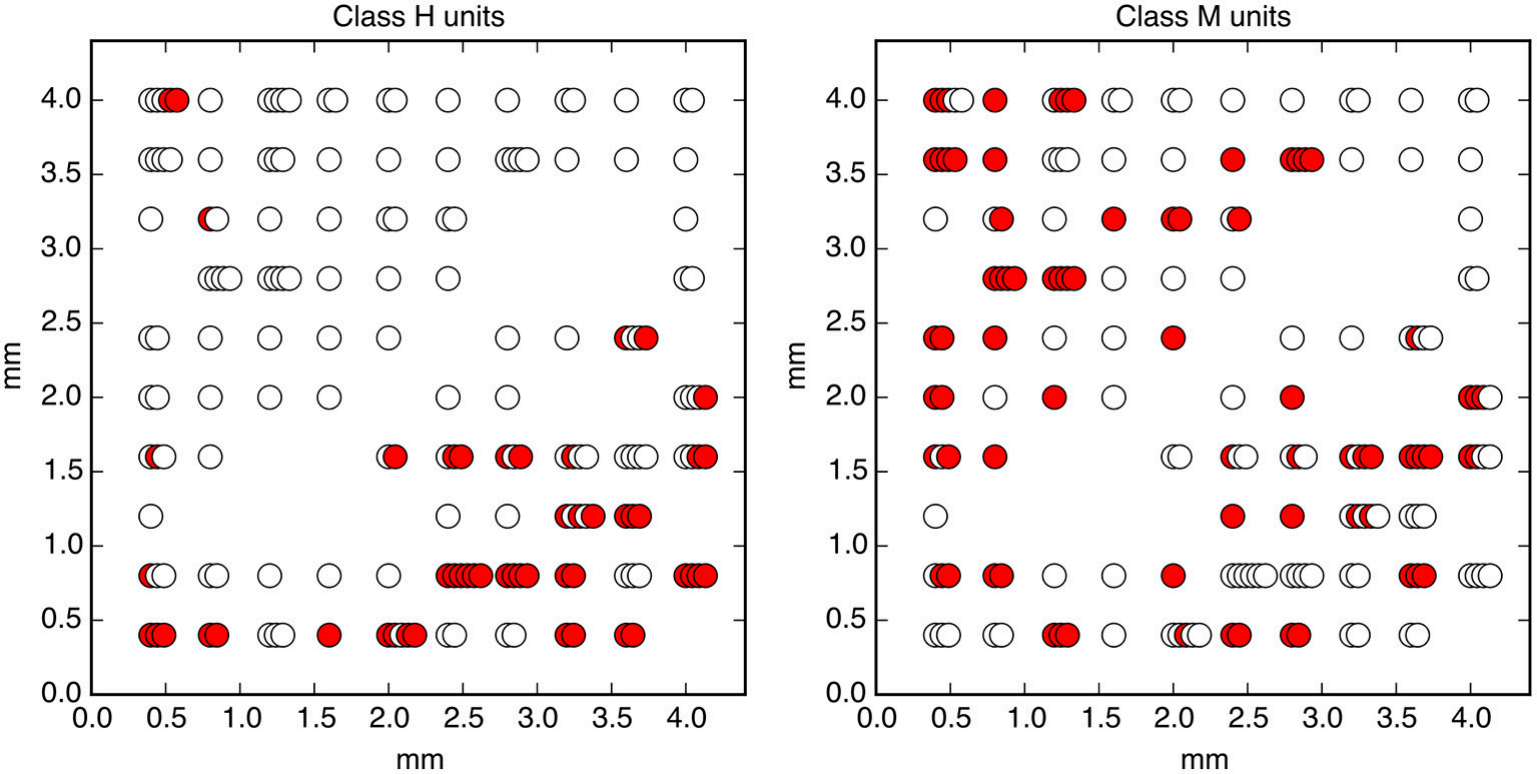
The physical locations of measured neural units. Colored circles indicate units that have significant mutual information with the decision sometime before the go cue (Class H, left) and only after the go cue (Class M, right). Stacked circles indicate multiple neural units detected by a single electrode.

Experimental data were provided by Roozbeh Kiani and William Newsome. Data were gathered in accordance with the recommendations of the National Institutes of Health Guides for the Care and Use of Laboratory Animals. The protocol was approved by the Stanford University Animal Care and Use Committee (IACUC number 9720). The data is a subset of that described in Kiani et al. (2014, 2015). Details of the stimulus and eye monitoring method are explained in those references. Neural spiking data consists of spike times measured at a resolution of 1/30 ms for 169 neural units using a 4 mm × 4 mm multielectrode array (see Figure 10). Data includes 1,778 trials taken from one animal in one recording session, with the signed coherence of the stimulus for each trial chosen at random from the set {−0.32, −0.16, −0.08, −0.04, −0.02, −0.01, 0, 0.01, 0.02, 0.04, 0.08, 0.16, 0.32}. Other task data for each trial include the direction of the decision saccade as well as the times of the onset of the stimulus, end of stimulus, go cue, and response saccade.

### 7.2. Dynamic, stochastic, distributed decision-making model

In a continuous-time model of neural activity (Hopfield, 1984; Beer, 1995), the time derivative of the state *x*_*i*_ of synapse *i* is the sum of external input *s*, leak current proportional to *x*_*i*_, and input from other neurons in the system. Input from each other neuron is assumed to be proportional to its current firing rate *r*_*j*_, which is a sigmoidal function of its current state: *r*_*j*_ = *g*(*x*_*j*_). This produces:

(5) $$\begin{array}{l} \tau_{i}\frac{dx_{i}}{dt}=s_{i}-x_{i}+\underset{j\neq i}{\sum }c_{ij}g_{j}\left(x_{j}\right), \end{array}$$

where the timescale of the cell returning to equilibrium in the absence of other signals is set by τ_*i*_. For simplicity, we assume complete homogeneity, with all-to-all synaptic connections of strength *c*, constant individual timescales τ_*i*_ = τ, and *g*_*i*_(*x*_*i*_) = *r*_*i*_ = tanh(*x*_*i*_):

(6) $$\begin{array}{l} \tau \frac{dx_{i}}{dt}=s-x_{i}+c\underset{j\;\neq \;i}{\sum }\tanh\left(x_{j}\right). \end{array}$$

Adding a Gaussian noise term ξ, with $\langle \xi \left(t_{0}\right)\xi \left(t_{1}\right)\rangle =\delta \left(t_{1}-t_{0}\right)N\left(0,\Gamma^{2}\right)$, we obtain Equation (1):

(7) $$\begin{array}{l} \tau \frac{dx_{i}}{dt}=s-x_{i}+\xi +c\underset{j\;\neq \;i}{\sum }r_{j}. \end{array}$$

The timescale τ is set at 10 ms to match the order of magnitude of the characteristic timescales of synaptic receptors (Moreno-Bote and Parga, 2010).

Importantly, we expect the qualitative features of the relationship between timescales and redundancy to be insensitive to the exact form of the dynamics. These features are produced near any collective transition displaying critical slowing down, as described below.

As the strength *c* of recurrent excitatory interactions increases, there is a transition from one stable state at *r*_*i*_ = 0 to two stable states at $r_{i}=\pm r^{*}$. Without noise (Γ = 0) and assuming identical individuals *r*_*i*_ = *r*, the dynamics becomes one-dimensional:

(8) $$\begin{array}{l} \tau \frac{dx}{dt}=s-x+c\left(N-1\right)\tanh x. \end{array}$$

As can be seen by measuring the stability of the *x* = 0 state (taking a derivative of Equation 8 with respect to *x*), this transition (bifurcation) is controlled by the degree of recurrent excitation $\bar{c}=c\left(N-1\right)$, with the transition occurring at $\bar{c}={\bar{c}}^{*}=1$. Above the instability transition and neglecting noise, the final state retains indefinitely a perfect memory of the sign of the input *s*. Furthermore, the characteristic timescale of moving away from *x* = 0, which sets the timescale for the decision process, is inversely related to the distance from this transition: $\tau_{\text{decision}}=\tau /\left(\bar{c}-1\right)$. When noise is added (Γ > 0), the attractors are destabilized, but we expect equivalent stability to be regained by moving to larger $\bar{c}$. This dependence of ${\bar{c}}^{*}$ on Γ is demonstrated in Figure 7.

The remaining parameters of the model can be set by matching the qualitative characteristics of informational dynamics observed in the data (see Figures 3, 4). First, the degree of recurrent excitation is set to roughly match the timescales with which information increases in Phases I and II: with no noise, ${\bar{c}}_{I}=1.1$ corresponds to accumulation on the scale of 100 ms in Phase I, and $\bar{c}=1.5$ to a faster timescale of 20 ms in Phase II.

**Figure 11:**
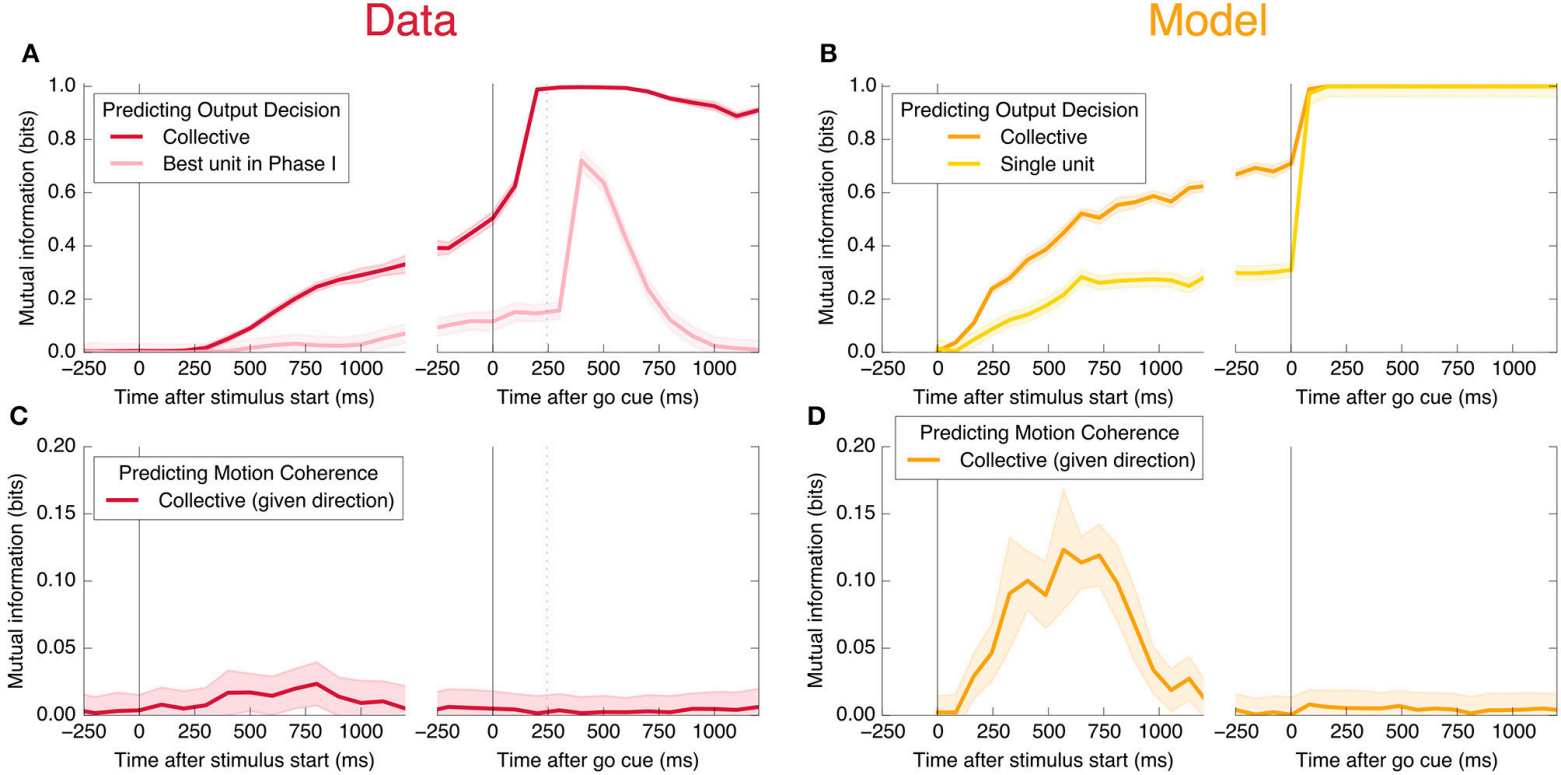
Mutual information with respect to the output decision, an information theoretic measure of the predictive power curves. Here we plot the mutual information with respect to the output decision **(A,B)** and motion coherence **(C,D)**. Note the different vertical scale in **(C,D)**. Trials are split into two equal and randomly selected halves, with one half treated as in-sample data used to calculate quantities for LDA classification, and the second half treated as out-of-sample data whose firing rates are used to make predictions.

Second, because the dynamics is only slightly perturbed by the input (Figure 11C), we next set *s* = 0 and match the behavior of information redundancy (Figure 4A) solely by varying the total number of model neurons *N*, the number of measured neurons *N*_measured_, and the amount of noise Γ. As *N* becomes larger, we expect that a larger Γ will produce the same dynamics and variance along the decision dimension $\vec{v}$, while corresponding to larger noise and thus less information carried by individuals.^3^ We find that *N* = 500, *N*_measured_ = 5, and Γ = 0.16 reproduces the qualitative features of the two phases. We do not attempt here to estimate the number of neurons truly involved in the decision-making process; this will require a more sophisticated model than presented here. Specifically, this model does not capture the strong heterogeneity that exists even within the defined cell classes, which will be important to estimating the actual number of required cells.

Finally, we set the form of the external input *s* representing transient sensory information. We assume that each cell receives input that is proportional to the signed coherence of the visual stimulus (with sign determined by the dominant direction of motion): *s* = *s*_*c*_ζ for coherence ζ. The signal is applied for the duration of the visual stimulus, 810 ms, after which it is set to zero. The proportionality constant *s*_*c*_ is set by roughly matching the proportion of correct decisions as a function of coherence (Figure 8). The values of all model parameters are listed in Table 3.

**Table 3:** Stochastic dynamic model parameters used throughout the paper, unless otherwise specified.

τ	10 ms
*N*	500
*N*_measured_	5
*s*_c_	0.03
*c*_I_(*N* − 1)	1.1
*c*_II_(*N* − 1)	1.5
Γ	0.16

*In Figure 7, N_measured_ = 25*.

We emphasize that there are very likely other sets of parameters that equally well match the qualitative features of the data, so the values of the individual parameters are not meant to represent best choices to be used in other contexts. For instance, moving along the transition line in Figure 7 produces largely equivalent behavior, demonstrating that distance from the point of instability is most important. We use the model not to infer specific parameters for individual level behavior, but rather (1) to show that it is possible to reproduce the behavior seen in both temporal phases using a simple model, and (2) to demonstrate the close relationships among distance from instability, timescale of accumulation, and redundancy of representation.

The model simulation recapitulates the timing of trials. In Phase I, with $\bar{c}={\bar{c}}_{I}$ and initial conditions *r*_*i*_ = 0 ∀*i*, the stimulus is presented for 810 ms, followed by an 810 ms delay period^4^ during which *s* = 0. The signed coherence takes values {−0.32, −0.16, −0.08, −0.04, −0.02, −0.01, 0, 0.01, 0.02, 0.04, 0.08, 0.16, 0.32}, with each signed coherence simulated 140 times for a total of 1820 simulation trials.^5^ Phase II consists of two more 810 ms time periods with increased $\bar{c}={\bar{c}}_{II}$, with initial conditions given by the final timestep of Phase I. Integration is performed using straightforward Euler timesteps, with 500 timesteps per 810 ms time period. Decreasing the step size by a factor of two does not qualitatively affect the results.

### 7.3. Details about mutual information

The mutual information is a standard measure of dependence in information theory. Calculating mutual information between random variables *A* and *B* begins with estimating the probabilities of all possible pairs of states of *A* (with states *a*_1_, *a*_2_, …) and *B* (with states *b*_1_, *b*_2_, …). Call these probabilities *P*(*a*_1_, *b*_1_), *P*(*a*_1_, *b*_2_), *P*(*a*_2_, *b*_1_), …. The marginal entropy *S*(*B*) measures the number of bits^6^ necessary to communicate the state of *B* without knowing the state of *A*. This marginal entropy $S\left(B\right)=-\sum_{j}P\left(b_{j}\right)\log P\left(b_{j}\right)$, where $P(b_{j})=\sum_{i}P\left(a_{i},b_{j}\right)$ sums over the unknown state of *A*. Similarly, $S(A)=-\sum_{i}P(a_{i})\log P(a_{i})$ with $P(a_{i})=\sum_{j}P(a_{i},b_{j})$. Finally, the joint entropy measures the number of bits necessary to describe the combined state of *A* and *B*: $S(A,B)=-\sum_{i}\sum_{j}P(a_{i},b_{j})\log P(a_{i},b_{j})$, and the mutual information is given by the difference of the total possible entropy of the paired data and its actual entropy:

(9) $$\begin{array}{l} I\left(A,B\right)=S\left(A\right)+S\left(B\right)-S\left(A,B\right). \end{array}$$

Intuitively, *I*(*A, B*) measures the reduction of uncertainty about the state of *B* when given the state of *A*, and vice versa.

### 7.4. Lower bound on mutual information via LDA

Estimating the mutual information between the collective firing pattern of the measured neurons and a behavioral variable requires estimating the entropy of each (Equation 9). The entropy of the neural firing is difficult to calculate because it has many dimensions and thus many possible states. ^7^

If we hypothesize a specific encoding, however, we can use the Data Processing Inequality to produce a lower bound on the mutual information. LDA constitutes such a hypothesis; by transforming the neural rate data $\vec{r}$ at a given time into a single continuous variable $v=\vec{v}\cdot \vec{r}$ (with $\vec{v}$ given by Equation 2), the mutual information of *v* with the variable in question provides a lower bound on the true mutual information.

In Figure 11, to estimate the entropy of the LDA projection *v* used to calculate the lower bound on the CMI, we bin values for *v* into *N*_*B*_ equally-spaced bins (with *N*_*B*_≪*N*_*s*_) and use the NSB method (Nemenman et al., 2004, 2002) on this reduced space. We choose *N*_*B*_ = 30 by increasing *N*_*B*_ until the mutual informations shown in Figure 11 saturate.

### 7.5. Details about Figure 3

Neural rates are calculated using a bin width of 200 ms for measuring information about the decision (Figure 3) and 500 ms for measuring information about the coherence of the stimulus (Figure 6). “Best unit in Phase I” refers to the unit with largest average mutual information in 200 ms time bins in Phase I (in the 2000 ms before the go cue). Data is split randomly into an equal number of in- and out-of-sample trials; we plot the resulting mean (line) and standard deviation (shaded area) over 20 of these random partitions in the case of predicting the output decision, and 100 partitions in the case of predicting motion coherence.

To test the amount of information present about coherence of the input, we split the trials into “strong coherence” trials (coherence value equal to 0.08, 0.16, or 0.32; 820 trials; relative frequency 0.461) and “weak coherence” trials (coherence value equal to 0, 0.01, 0.02, or 0.04; 958 trials; relative frequency 0.539). Mutual information and out-of-sample prediction are measured with respect to the binary classification of strong vs. weak, leading to a maximum possible mutual information of −0.461log0.461−0.539log0.539 = 0.996 bits. Previous studies have found that variation in firing rates is opposite in sign depending on the output direction (Shadlen and Newsome, 2001). For this reason, without knowing the eventual output, differences in firing rates due to stimulus coherence tend to be invisible using the linear LDA encoding. The coherence LDA calculations and predictions are thus performed separately in the two output direction conditions and then averaged; this corresponds to the task of predicting the coherence of the signal given both neural firing rates and the eventual output direction.

In Figure 11, we plot the mutual information measures corresponding to the predictive power plotted in Figure 3.

### 7.6. Details about Figure 4

To measure the number of units needed to reach a certain predictive performance, we add neurons one at a time ordered by their individual mutual information with the output at the given time (calculated with bin width 200 ms). Plotted lines indicate means and shaded regions indicate standard deviations over 20 realizations of in-sample and out-of-sample partitioning, as described above with regard to Figure 3.

**Figure 12:**
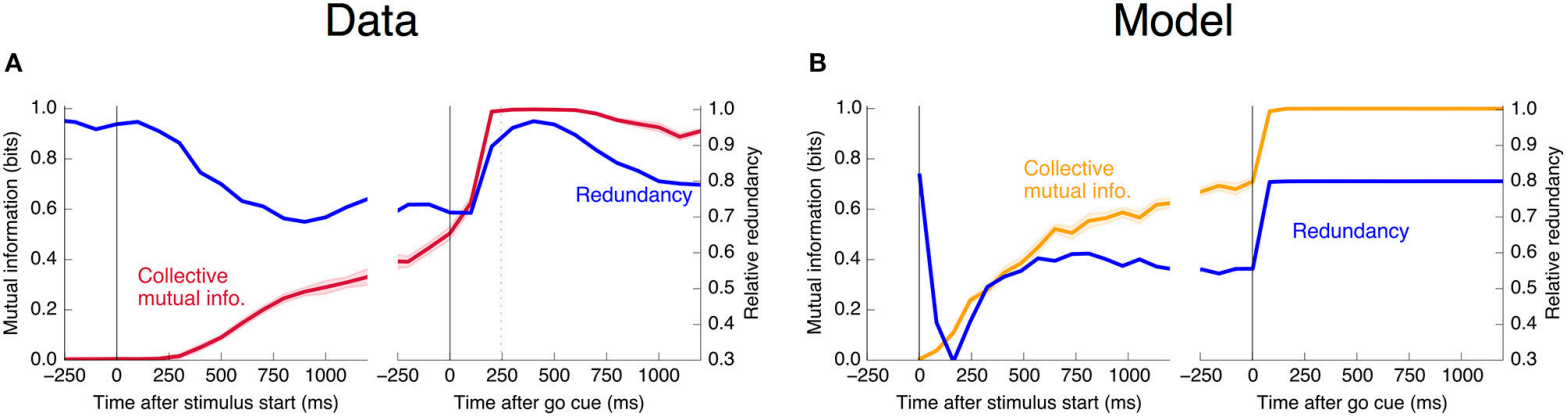
Informational redundancy decreases during Phase I and increases during Phase II. Redundancy is measured by Equation 10, with similar dynamics displayed in **(A)** the neural data and **(B)** the simple rate model.

Though it is intuitive, our measure of the number of individual units needed to reach the collective performance may not be ideal for future experiments in that we expect it to become uninformative for large *N* when individual neural behavior is heterogeneous. If there is some small fraction of neurons that are individually very informative (as was found in Shadlen and Newsome, 2001), then the number of units needed to reach the collective performance will always approach 1 as *N* becomes large. Alternatively, we can use an information theoretic measure of redundancy that also takes into account the number of very informative units. We use here the relative redundancy:

(10) $$\begin{array}{l} R=1-\frac{I\left(\text{all units},\text{ decision}\right)}{\sum_{i}I\left(r_{i},\text{ decision}\right)}, \end{array}$$

estimating the collective and individual mutual informations in the same way as in Figure 11. This redundancy is plotted as a function of trial time in Figure 12. The story is the same as that told by Figure 4: Units become more redundant in the information they encode about the output near the time of the saccade in Phase II.

**Figure 13:**
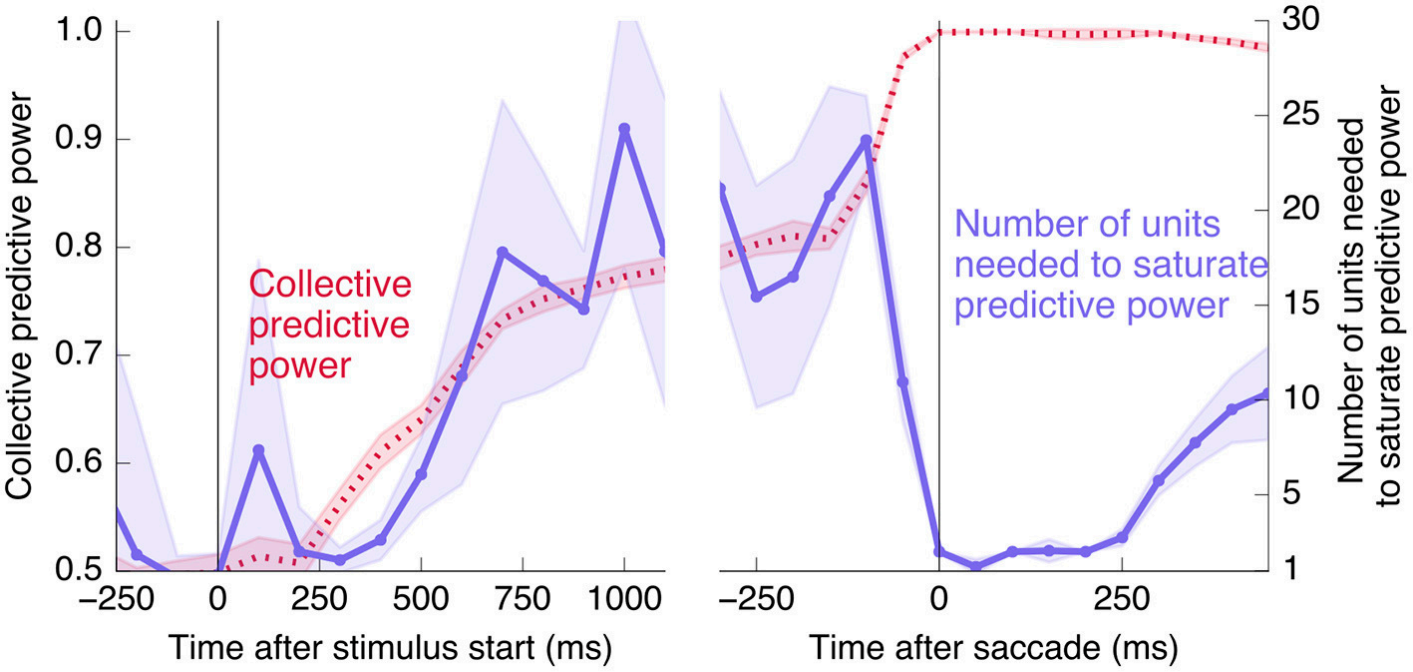
Changes to distributedness happen prior to saccade. The left plot is the same as Figure 4A, and the right half shows the same calculation using data aligned at the saccade start instead of the go cue (and using 100 ms time bins). Note that the decrease in the number of units needed to saturate predictive power begins before the saccade.

Finally, we would like to test directly the extent to which the change in redundancy happens before or after the saccade. Aligning by the saccade time instead of the go cue, and using a smaller time window of 100 ms for finer temporal resolution, produces the right half of Figure 13. We see that the abrupt increase in collective information and decrease in distributedness begins before the saccade. This rules out an interpretation in which the change is caused only by visual feedback coming from the execution of the eye movement. The datapoint at *t* = 0 corresponds to a time window from 50 ms before to 50 ms after the saccade, during which it is unlikely that any visual feedback signal has reached the measured neurons. Even ignoring this datapoint, however, the number of units needed to saturate predictive power decreases significantly before the saccade: Saturation using rates in the 100 ms prior to the go cue requires 23 ± 5 units, whereas the 100 ms prior to the saccade requires 11 ± 2 units (and a 100 ms window 250 ms after the saccade requires 2.8 ± 0.4 units, in each case averaged over 20 in- and out-of-sample shuffles, as in Figure 4).

### 7.7. “Wisdom of the crowd” argument

Besides avoiding premature saturation, another argument for decreased interactions during decision-making comes from a “wisdom of the crowd” argument, in which noise in individual decisions is best removed from an average by having individuals cast independent votes. We do not focus on this explanation because the magnitude of the effect in general depends on the specifics of the interactions, and for some cases (e.g., each individual moves their opinion closer to the average of individuals it interacts with) has no effect on the accuracy of the decision.

For fixed means and variances, it is true that having noise correlations that are the same sign as signal correlations leads to worse performance (as in Jeanne et al., 2013, originally explored in Abbott and Dayan, 1999). Yet this is not easily connected to optimizing interactions for decision-making, because changing interactions in a dynamical context does not typically leave means and variances fixed.

## 8. Ethics statement

This study was carried out in accordance with the recommendations of the National Institutes of Health Guides for the Care and Use of Laboratory Animals. The protocol was approved by the Stanford University Animal Care and Use Committee (IACUC number 9720).

## Author contributions

BD, JF, and DK conceptualized the study and wrote the paper. BD performed the data analysis.

### Conflict of interest statement

The authors declare that the research was conducted in the absence of any commercial or financial relationships that could be construed as a potential conflict of interest.

## Footnotes

^1^This logic can likely be generalized to decision-making involving more than two options by using Hopfield networks that involve both positive and negative interactions. An analogous parameter to *c* in that case is the gain λ in Hopfield (1984).

^2^Too far below the threshold, information is quickly forgotten, and too far above the threshold, a decision is made prematurely.

^3^The exact scaling with *N* is not obvious in this strongly interacting system.

^4^The duration of this delay period was randomized in the experiment, taking values between 300 and 1500 ms, in order to avoid anticipation of the go cue. We do not include this variation in our simulations as we do not expect it to affect the results—the behavior of the model during the delay period is largely static.

^5^In the experiment, the coherence level was chosen at random from this set for each trial, so the number of trials per coherence was not precisely uniform.

^6^Measuring entropy in bits corresponds to taking logarithms with base 2 in the following formulas.

^7^Even if we bin the spikes into time intervals and create binary data, to guarantee a good estimate of the entropy, we need a number of samples $N_{s}\gg 2^{N}$ (or perhaps $N_{s}\gg \sqrt{2^{N}}$ using a method such as NSB). With 1778 trials, we can reliably estimate the entropy only if *N* < log_2_*N*_*s*_ = 10.8 (or perhaps *N* < 2 log_2_*N*_*s*_ = 21.6), so using all units, even restricted to one of our defined classes, is not possible.
